# Assessment of the range of the HIV-1 infectivity enhancing effect of individual human semen specimen and the range of inhibition by EGCG

**DOI:** 10.1186/1742-6405-9-2

**Published:** 2012-01-19

**Authors:** Philip Hartjen, Sebastian Frerk, Ilona Hauber, Verena Matzat, Adriana Thomssen, Barbara Holstermann, Heinrich Hohenberg, Wolfgang Schulze, Julian Schulze zur Wiesch, Jan van Lunzen

**Affiliations:** 1 Infectious Diseases Unit, I. Department of Internal Medicine, University Medical Center Hamburg-Eppendorf, Martinistrasse 52, 20251 Hamburg, Germany; 2 Department of Andrology, University Medical Center Hamburg-Eppendorf, Martinistrasse 52, 20251 Hamburg, Germany; 3 Heinrich Pette Institute - Leibniz Institute for Experimental Virology (HPI), Martinistrasse 52, 20251 Hamburg, Germany

**Keywords:** Semen, SEVI, EGCG, HIV transmission, microbicide

## Abstract

Recently, it has been shown that human ejaculate enhances human immunodeficiency virus 1 (HIV-1) infectivity. Enhancement of infectivity is conceived to be mediated by amyloid filaments from peptides that are proteolytically released from prostatic acid phosphatase (PAP), termed Semen-derived Enhancer of Virus Infection (SEVI). The aim of this study was to test the range of HIV-1 infectivity enhancing properties of a large number of individual semen samples (n = 47) in a TZM-bl reporter cell HIV infection system. We find that semen overall increased infectivity to 156% of the control experiment without semen, albeit with great inter- and intraindividual variability (range -53%-363%). Using transmission electron microscopy, we provide evidence for SEVI fibrils in fresh human semen for the first time. Moreover, we confirm that the infectivity enhancing property can be inhibited by the major green tea ingredient epigallocatechin-3-gallate (EGCG) at non-toxic concentrations. The median inhibition of infection by treatment with 0.4 mM EGCG was 70.6% (p < 0.0001) in our cohort. Yet, there were substantial variations of inhibition and in a minority of samples, infectivity enhancement was not inhibited by EGCG treatment at all. Thus, topical application of EGCG may be a feasible additional measure to prevent the sexual transmission of HIV. However, the reasons for the variability in the efficacy of the abrogation of semen-mediated enhancement of HIV-1 infectivity and EGCG efficacy have to be elucidated before therapeutic trials can be conducted.

## Background

HIV-infection is an imminent health issue, with an estimated 33 million individuals infected worldwide according to UNAIDS [1]. Globally, most HIV infections occur by heterosexual transmission (for review see [2]). Sexual HIV-1-transmission depends on viral and multiple host factors that altogether have not been entirely unraveled [3], and a direct role of semen has been described by several groups (reviewed in [4]).

Recently, it has been reported that human ejaculate acts as a potent enhancer of HIV infectivity [5]. This enhancement of infectivity is mediated by a factor, termed Semen-derived Enhancer of Virus Infection (SEVI) [5]. SEVI was identified to be a peptide fragment of the semen marker prostatic acidic phosphatase (PAP) that, upon proteolytic release, forms amyloid fibrils. These fibrils capture HIV virions and direct them to target cells, where they facilitate the fusion of virus and host cell [6].

Interestingly, it has been previously demonstrated that epigallocatechin-3-gallate (EGCG), the major active constituent of green tea, can inhibit the infectivity enhancing effect of SEVI, possibly by interference with de novo SEVI formation or by degradation of present preformed PAP-derived amyloid fibrils [7]. This observation may be important for possible application of EGCG in microbicidal vaginal and rectal gels that could reduce HIV transmission rates [8,9].

To date, published studies on SEVI (and EGCG) are predominantly based on *in vitro *experiments carried out either with pooled human semen or with fibrils formed from synthetic PAP-fragment peptides (PAP248-286) [5,7]. Hence, the aim of the current study was to determine the range and variability of the HIV-1 infectivity enhancing properties and the effect of EGCG on infectivity enhancement within a cohort of individual human semen samples, and to describe the clinical semen characteristics that are potentially associated with the augmentation of HIV-1-infectivity. In addition, this is the first study that provides evidence for the presence of SEVI fibril structures in human semen by transmission electron microscopy.

## Results

### Individual semen samples enhance HIV-1 infection

**Figure 1:**
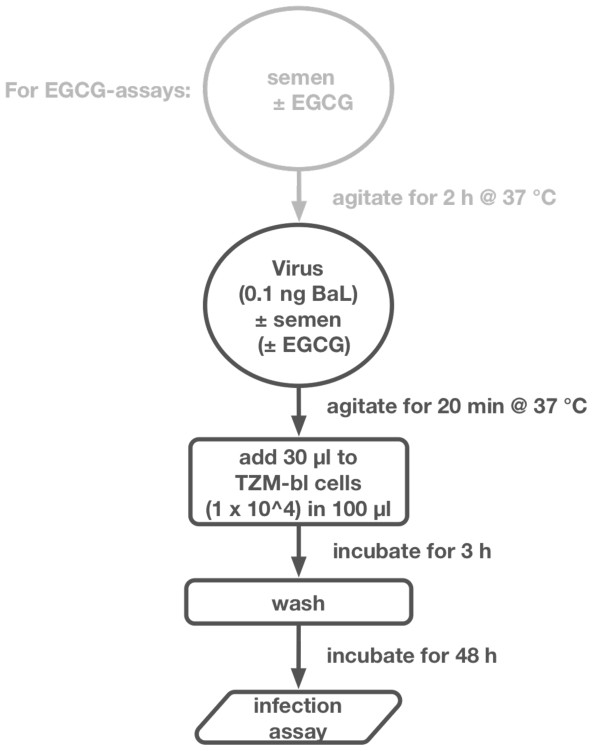
**Schematic outline of the experimental procedure**.

47 individual semen samples, originating from HIV-1 negative, clinically infertile men and healthy donors were analyzed. Full spermiograms according to the criteria of the WHO (1999) [10] and routine laboratory parameters were determined for all samples. All donors gave written informed consent for this study that was approved by the local ethics committee. To assess the HIV-1-infectivity enhancing properties of all individual semen samples, TZM-bl reporter cells were infected with HIV-1 BaL that was preincubated in the presence or absence of semen. Infection levels were then determined by measuring luciferase activity. A detailed overview of the experimental design is depicted in Figure 1.

**Figure 2:**
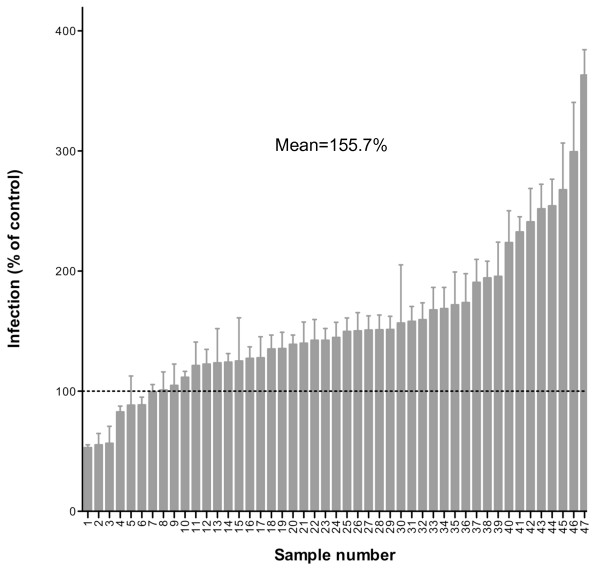
**HIV-1 infectivity enhancing properties of human semen samples**. 47 semen samples were analyzed for HIV-1-infectivity modulating effects. Results are arranged by infectivity enhancing potency. Semen was used in a 1:16 dilution during preincubation. The Y-axis denotes the infection rate relative to control infections performed without semen (depicted as dotted line) as percentage. Shown are mean values of quintuplicate infections. Error bars represent the standard error of the means.

Our first major finding is, that the majority of semen samples enhance HIV-1 infectivity (Figure 2). However, there was considerable heterogeneity in the infectivity enhancing properties, and a minority of semen samples (6/47) even caused a slight decrease in HIV-1 infectivity. On average, the infection rate was enhanced to 155.7% (range -53%-363%) of the control experiment in absence of semen.

**Figure 3:**
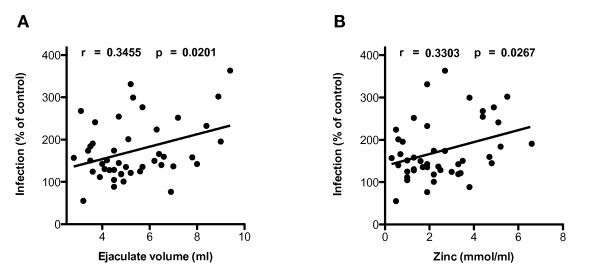
**Infectivity enhancing potential of semen samples correlates with clinical semen parameters**. Ejaculate volume **(A) **and zinc concentration **(B) **were plotted against the enhancement of HIV-1 infectivity. Correlations were determined using the two-tailed Pearson correlation coefficient.

All clinical semen parameters were tested with respect to the infectivity enhancing properties and moderate correlations with two clinical semen-parameters were noticed (Figure 3): The ejaculate volume (r = 0.3455, p = 0.0201), and the concentration of zinc (r = 0.3303, p = 0.0267) correlated positively with enhancement of infection at univariate analysis. Of note, the ejaculate volume did not correlate with days of sexual abstinence. Other analyzed semen parameters (proportions of mobile and immobile spermatozoa, number of round cells, pH, concentrations of fructose, zinc, citrate and carnitine) did not correlate significantly with the enhancement of HIV-1 infection, although there was a trend towards a positive correlation with the concentration of citrate (r = 0.2510, p = 0.0963). It will be important to test in subsequent studies, whether any of these parameters could have some influence on amyloid fibrillogenesis in semen or the rate of SEVI-degradation. The minority of samples that inhibited infectivity did not share an obvious clinical characteristic or parameter.

### Semen contains fibrils resembling synthetic SEVI fibrils

**Figure 4:**
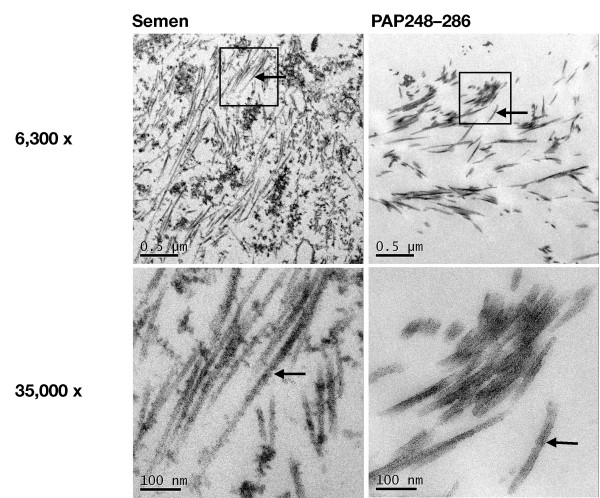
**Transmission electron microscopy analysis of semen and synthetic SEVI in a closed system**. A fresh semen sample and a solution of chemically synthesized SEVI (PAP-fragment PAP248-286) were analyzed as described in the methods section. The bottom images depict a detail (magnification: 35,000 ×) of the above electron micrographs (magnification: 6,300 ×). The semen sample shows a high density of fibrils with lengths ranging from 300 nm to about 3 μm, that exhibit a clear resemblance to the fibrils formed from chemically synthesized SEVI (PAP-fragment PAP248-286). The synthetic SEVI fibrils frequently run out of pane due to the embedding angle. Arrows indicate typical fibril structures.

It has been demonstrated that the chemically synthesized PAP-fragment PAP248-286 forms amyloid fibrils (SEVI fibrils) [5], which have been visualized by transmission electron microscopy (TEM) [7]. However, semen samples to date have not been analyzed for the presence of fibrils. We therefore subjected a human semen sample and an agitated solution of PAP248-286 containing SEVI fibrils to TEM. Inspection of the corresponding micrographs revealed the presence of large fibrils in the semen sample (Figure 4). These fibrils range in size from 300 nm to about 3 μm and closely resemble synthetic SEVI-fibrils formed from PAP248-286.

### Semen-mediated enhancement of HIV-1 infectivity is abrogated by EGCG at non-toxic concentrations in the large majority of semen samples

**Figure 5:**
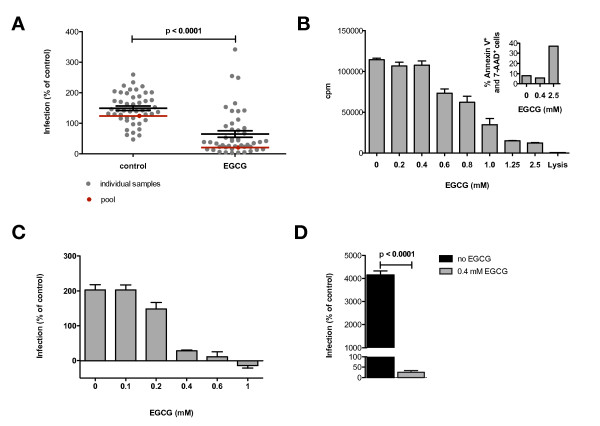
**EGCG abrogates semen-enhanced viral infection and the effect of synthetic SEVI at nontoxic concentrations**. **A**) 47 individual semen samples were preincubated separately and pooled in presence or absence of 0.4 mM EGCG and subjected to the infection assay as described in the methods section. Semen was used in a 1:16 dilution during preincubation. The Y-axis denotes the infection rate as percent in relation to control infections performed without semen. Data points represent mean values of quintuplicate infections, black lines show the mean values for all samples and standard error of the means. Red lines show the results for pooled semen. The indicated p-value is the result of a paired, two-tailed Student's *t*-test analysis. **B) **Cellular viability in presence of EGCG was tested in a [^3^H]-thymidin incorporation assay. Data is expressed as counts per minute, shown are mean values of quintuplicate measurements. Error bars represent the standard error of the means. The insertion figure shows the results of a flow cytometry-based apoptosis/necrosis assay utilizing Annexin-V and 7-AAD. The Y-axis denotes the percentage of Annexin V^+^, 7-AAD^+ ^and double positive cells. **C) **A random individual semen sample was subjected to the infection assay as described above in presence of increasing EGCG concentrations. **D) **Synthetic SEVI (250 μg/ml) in presence or absence of 0.4 mM EGCG was subjected to the infection assay as described above. The indicated p-value is the result of a paired, two-tailed Student's *t*-test analysis.

In a next step, inhibition of the infectivity enhancing effect of semen by preincubation with EGCG was tested within the entire cohort (Figure 5A). An EGCG concentration of 0.4 mM was chosen to rule out toxicity in our assay (Figure 5B). We find, that for the majority of semen samples, the augmentation of HIV-1 infection was indeed drastically inhibited by treatment with EGCG. This effect was concentration dependent (Figure 5C). Overall, EGCG treatment decreased the infection rate by a median of 70.6% (p < 0.0001). However, we observed substantial heterogeneity for the inhibition of semen-mediated enhancement of HIV infection by EGCG (range -111%-98%). EGCG had an inhibitory effect on only 41 of the 47 samples.

To validate the inhibitory effect of EGCG, we also subjected a pool of all 47 analyzed semen samples to the infection assay in presence or absence of EGCG. Pooled semen increased the infection rate to 124% and EGCG treatment lead to a complete abrogation of the infectivity enhancement by pooled semen. Of note, the rate of HIV-1 infection enhancement by pooled semen samples was slightly lower than the mean of measurements for the individual samples.

Direct EGCG toxicity at doses used to block enhancement of infectivity (0.4 mM) was ruled out in a [^3^H]-thymidin incorporation assay and in a flow cytometry-based apoptosis/necrosis assay utilizing Annexin-V and 7-AAD (Figure 5B).

We next wanted to verify the efficacy of EGCG treatment for synthetic SEVI. Synthetic SEVI enhanced HIV-infectivity drastically, leading to a 40-fold (4000%) increase in the infection rate in our assay when applied in a concentration of 250 μg/ml. Treatment with 0.4 mM EGCG completely abolished this effect (Figure 5D).

**Figure 6:**
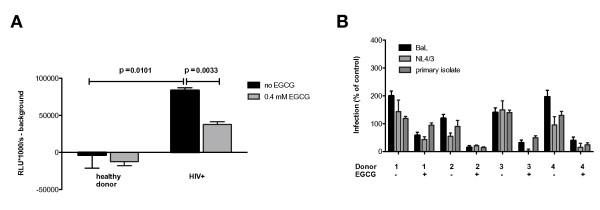
**EGCG effectively inhibits semen-enhanced viral infection of autologous virus/semen and R5- as well as X4-tropic viruses**. **A**) Semen samples from a healthy donor and an HIV-1-positive patient with high viremia were preincubated in presence or absence of 0.4 mM EGCG and subjected to the infection assay as described in the methods section. Semen was used undiluted during preincubation. Columns represent luciferase activity minus background (luciferase activity in control experiments performed without semen) expressed as RLU/s*1000. Error bars represent the standard error of the means. The indicated p-values result from unpaired, two-tailed Student's *t*-test analysis. Significantly higher luciferase activity in cells incubated with semen from the HIV-patient than in cells incubated with semen from the healthy donor demonstrates successful infection. **B) **To analyze semen-mediated enhancement of HIV-1 infectivity and its inhibition by EGCG with regards to different virus strains, three different HIV-1 strains were deployed: BaL (R5-tropic), NL4/3 (X4-tropic) and a primary HIV-1B isolate. Four individual semen samples were subjected to the infection assay described above in presence or absence of 0.4 mM EGCG.

To test the efficacy of EGCG under experimental conditions that better reflect the *in vivo *situation, we incubated TZM-Bl cells directly with semen from an HIV-positive, highly viremic donor in presence or absence of 0.4 mM EGCG (Figure 6A). This approach resulted in successful infection and EGCG-treatment led to a 55% reduction of the infectivity of autologous virus/semen (p = 0.0033).

To elucidate whether semen-mediated enhancement of HIV-1 infectivity and its inhibition by EGCG was dependent on certain viral strains and/or viral coreceptor tropism, a panel of three different HIV-1 strains and four individual semen samples were tested (Figure 6B). We observed a similar pattern for all three viral strains tested. Measured values for X4-tropic NL4/3 and primary HIV-1B isolate in absence of EGCG differ by 35% and 25% respectively from the infectivity enhancement observed for R5-tropic BaL. EGCG-treatment reduced the infectivity of all analyzed viruses in presence of each of the investigated semen samples, albeit to varying degrees. The results of these experiments confirm the interindividual spread of the infectivity enhancing effect in semen and its inhibition by EGCG for X4- and R5-tropic laboratory strains and for a primary HIV-1B isolate.

### The infectivity enhancing potential and clinical parameters of semen vary between different samples from identical donors

**Figure 7:**
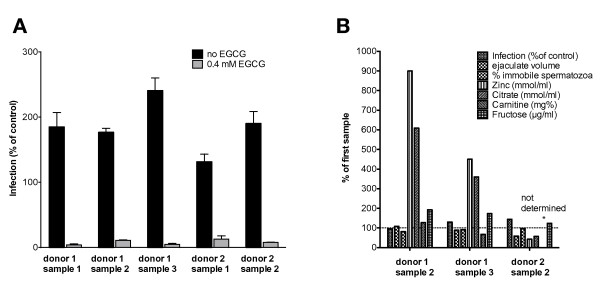
**Variation of infectivity enhancing potential and clinical parameters of semen between different samples from identical donors**. Semen samples from two healthy donors were analyzed longitudinally. **A) **Infectivity modulation in relation to control infections without semen in experiments in absence (black bars) or presence (grey bars) of EGCG as percentage. Shown are mean values of quintuplicate infections and standard error of the means. **B) **Changes of infectivity modulation and clinical semen parameters in semen samples from the same donors. The Y-axis denotes values for follow-up samples relative to baseline values.

Longitudinal analysis showed that the infectivity enhancing effect varies within semen samples originating from the same donors. We observed variation up to 45% (Figure 7A and 7B). There was also substantial variation in the composition of the samples (Figure 7B). Concentrations of zinc and citrate differed up to 900% and 600% respectively between samples from identical donors.

## Discussion

The aim of this study was to analyze the infectivity enhancing properties of a large number of individual human semen samples. We show that individual semen samples overall significantly enhance HIV-1 infection *in vitro*. We could also directly visualize fibrils in human semen, which resemble fibrils formed from synthetic PAP248-286 by transmission electron microscopy. For the majority of semen samples, the enhancement of HIV-1 infection can be drastically inhibited by treatment with non-toxic concentrations of EGCG, a small-molecule inhibitor of amyloid fibrillogenesis [11] (median inhibition = 70.6%, p < 0.0001). The efficacy of EGCG as an inhibitor of semen-mediated infectivity enhancement was confirmed for pooled semen, for autologous virus/semen from an HIV-positive, highly viremic donor and for synthetic SEVI.

Kim et al. recently demonstrated that HIV-1 infectivity enhancement by semen is highly heterogeneous [12]. Here we confirm and extend their finding that the ability of individual semen samples to enhance *in vitro* HIV-1 infection differs considerably. A minority of samples even inhibited infectivity in our experimental setup. We show that the HIV-1 infectivity enhancing potency is variable even in longitudinal semen samples originating from the same donor obtained at different time points. This finding underscores the high degree of heterogeneity and fluctuations of semen properties, which will pose a significant problem for future interventional trials.

We also used a panel of three different X4- and R5-tropic HIV-1 strains in our experiments, thereby confirming that the infectivity enhancing effect in semen is independent of the viral coreceptor tropism (Figure 6B). Synthetic SEVI enhanced HIV-1 infectivity about 25 fold stronger than semen in our experimental setup (Figure 5D). This does not contradict our results for semen, as the employed concentration of synthetic SEVI (250 μg/ml) was unphysiologic and much higher than the estimated concentration of SEVI in semen (35 μg/ml, as estimated by Münch et al. [5]). However, these results demonstrate the great potential of synthetic SEVI as an enhancer of *in vitro* lentiviral infection rates [13].

In contrast to our results, Kim et al. observed overall greater semen-mediated enhancement of HIV-1 infectivity (ranging from 2 to about 50-fold) [12], most likely due to differing experimental conditions and amounts of HIV-virions used for infection. We observed a strong dependence of the absolute HIV-infectivity enhancement on the amount of inoculum (semen-mediated enhancement is approximately threefold stronger when 20 pg BaL-p24 are used for infection instead of the utilized 100 pg). However, the experimental conditions for our study were chosen to ensure maximal test reliability and allow reproducible, consistent results.

In the above mentioned study, Kim et al. have demonstrated that semen-mediated HIV-1 infectivity enhancement correlates with SEVI/PAP248-286 levels measured by ELISA utilizing antisera from guinea pigs and rabbits [12]. Centrifugation through a 100-kDa-pore-size filter removed the entire virus enhancing activity and the reactivity to anti-SEVI antiserum, demonstrating that the enhancing factor has a molecular weight of > 100 kDa, which is in agreement with the fibril hypothesis of infectivity enhancement. A correlation between fibril abundance and HIV-1 infectivity enhancement was also demonstrated by Hauber et al. for synthetic SEVI fibrils, quantified by Congo red staining [7].

Possible causes for the observed heterogeneity of the HIV-infectivity enhancing potency of semen, besides the amount of SEVI fibrils present, could be the variable abundance of cationic polypeptides that inhibit HIV-1 infection [14] and/or naturally occurring proteases in semen. In a recent study, Martellini et al. show that human seminal plasma inhibits fibril formation of PAP248-286 and exhibits proteolytic activity that can inhibit the proviral activity of SEVI under certain conditions [15]. Interestingly, we found a positive correlation between HIV-1 infectivity enhancement and the concentration of zinc in semen by univariate analysis. The findings of Martellini et al. could offer an explanation for this correlation. As Zn^2+ ^exhibits inhibitory effects on semen proteases [16], it is intruiging to hypothesize that SEVI fibrils could be more stable in semen samples that contain high amounts of Zn^2+^, and thus tend to have a higher capacity to enhance HIV infectivity.

Our results confirm and extend the original findings by Hauber et al., who first described the inhibition of semen-mediated enhancement of HIV-infectivity by EGCG for a limited number of individual semen samples [7]. While it was thought that the SEVI inhibiting effect by EGCG is mainly mediated through its anti-fibrillogenic properties, we also observed semen-independent inhibition of HIV-infectivity at non-toxic EGCG concentrations. For some samples and for the pool of all samples, the infection rate in presence of EGCG and semen was reduced to below the infection rate of control experiments performed in absence of EGCG and semen. To elucidate this effect, we tested the direct antiviral activity of EGCG in absence of semen in our assay. In contrast to previous results by Hauber et al. with Jurkat 1G5 cells and HIV-1 NL4/3 [7], we observed a semen-independent inhibition of HIV-infectivity by EGCG. The presence of 0.4 mM EGCG in absence of semen resulted in 88.5% inhibition of HIV infectivity. Several other studies also found a direct inhibition of HIV-infectivity by EGCG, albeit in different cells [17-20]. This inhibition is thought to be mediated through direct interaction with the CD4 receptor and/or HIV-1 Env. A direct antiviral activity of EGCG has also been described for other viruses, namely herpes simplex virus (HSV) [21] and hepatitis C virus (HCV) [22]. Ciesek et al. recently demonstrated that EGCG is an inhibitor of hepatitis C virus entry [22]. Regardless of the mechanism, EGCG treatment effectively abolished enhancement of HIV-1 infectivity of the majority of individual semen samples, of pooled semen, and of synthetic SEVI in our experimental setup. Direct inhibition of the infectivity of HIV and other viruses by EGCG would be an additional advantage for its use in antiviral microbicides.

Clinically, the systemic administration of EGCG has been proven to be safe and well tolerated in several studies [23-26]. Topical application of EGCG has been tested in mice with no dermal toxicity detected after ointment application (up to 3% w/w) daily for 30 days [27]. 3% w/w EGCG corresponds to a concentration of 65.5 mM, more than 100-fold higher than the effective concentration used in our assay for inhibition of semen-mediated enhancement of HIV infection. Its safety and low cost together with its effects on other pathogens such as HSV [21] could make EGCG an attractive additional supplement for antimicrobial gels.

In agreement with Münch et al., it has to be pointed out that our results may underestimate the potency of SEVI for in vivo infectivity enhancement [5]. The quantity of HIV-1 virions transmitted during sexual intercourse is considerably lower than the amount of virions used in our assay, and the relative HIV-infectivity enhancement strongly depends on the amount of inoculum. Testing of semen-mediated enhancement of HIV-1 infection in animal models is warranted to clarify its role in sexual transmission of HIV-1.

In summary, we demonstrate for the first time that fresh human semen contains fibrils with clear resemblance to fibrils formed from synthetic SEVI. Moreover, we demonstrate that the semen-meditated enhancement of HIV-1 infectivity is highly variable and that EGCG can indeed effectively abrogate this activity at non-toxic concentrations in the majority of semen samples. Our study highlights the high degree of variation of HIV-1 infectivity enhancement by individual semen samples (as well as its inhibition by EGCG), even of longitudinal samples originating from identical donors. While EGCG holds potential as a possible microbicide, these variations have to be taken into account and further elucidated before therapeutic trials may be conducted.

## Methods

### Handling of semen and synthetic SEVI

All semen samples were liquefied for 30 min and kept frozen at -20°C until they were used in the experiments. PBS supplemented with 100 units/ml penicillin, 100 mg/ml streptomycin was used to dilute semen as indicated. A solution of chemically synthesized SEVI was prepared as described earlier [7]. Briefly, a 5 mg/ml solution of the peptide corresponding to amino acid residues 248-286 of PAP (EMBL accession no. AAB60640), in PBS was agitated at 37°C and 1, 200 rpm for 2-3 days (i.e. until the solution became turbid) to initiate fibril formation [7].

### Cell culture and HIV infection experiments

To analyze infectivity-modulating effects in semen, TZM-bl reporter cells, which can be infected with both R5- and X4-tropic virus and allow the quantification of HIV infection via an integrated, Tat-responsive HIV-1 LTR-luciferase reporter expression cassette [28,29] were employed. 10^4 ^cells were seeded in microtiter wells in a volume of 100 μl. After 24 h, cells were infected with HIV-1 (BaL [30], NL4/3 [31] or a primary HIV-1B isolate, corresponding to 100 pg p24 BaL) that was preincubated in the presence or absence of semen in the concentrations indicated in the figure legends with or without the addition of EGCG for 20 minutes. After 3 h at 37°C, the cells were washed and further cultured. At 48 h post infection, luciferase activity was determined. EGCG was obtained from Sigma Aldrich, Germany, and a stock solution of 10 mM was prepared in PBS and stored at -20°C. TZM-bl cells and HIV-1 isolates BaL and NL4/3 were obtained from the NIH AIDS Research and Reference Reagent Program.

### Analysis of cellular toxicity

Cellular viability in presence of EGCG was tested in a [^3^H]-thymidin incorporation assay and a flow cytometry-based apoptosis/necrosis assay utilizing Annexin-V and 7-AAD. For the [^3^H]-thymidin incorporation assay, 10^4 ^TZM-bl cells were seeded in microtiter wells in a volume of 100 μl. After 24 h, cells were incubated for 3 h in the presence of EGCG at indicated concentrations, washed and 0.5 μCi [^3^H]-thymidin was added. After further cultivation for 48 h, the cells were harvested on filters and [^3^H]-thymidin icorporation into DNA was determined by liquid-scintillation counting. For the Annexin-V/7-AAD Assay, 10^4 ^TZM-bl cells were seeded in microtiter wells in a volume of 100 μl. After 24 h, cells were incubated for 3 h in presence of EGCG in the indicated concentrations, washed and cultivated further for 48 h. Then, the cells were trypsinized and stained with 7-AAD and FITC-labeled Annexin V (both from BD Biosciences) according to the manufacturers protocol. Data were collected on a FACS Canto flow cytometer (BD Biosciences, Germany).

### Electron microscopy

For transmission electron microscopy analysis, semen and a solution of synthetic SEVI (5 mg/ml) were encapsulated in capillary microtubes and processed for ultrathin sectioning as described earlier [7,32].

### Statistical analysis

We determined the statistical significance of differences using Student's *t*-test analysis as indicated in the figure legends. Correlations were determined using the two-tailed Pearson correlation coefficient. Graphpad Prism version 5 was used for all calculations. For all analyses, p-values of less than 0.05 were considered significant.

## Competing interests

The authors declare that they have no competing interests.

## Authors' contributions

The work presented here was carried out in collaboration between all authors. JvL and JSzW defined the research theme. SF, PH, JSzW, JvL conceived and designed the study. JSzW provided most of the funding. SF, PH, IH, VM and AT carried out most of the laboratory experiments. SF, PH, JSzW and JvL analyzed the data and interpreted the results. PH and JSzW wrote the first draft. JVL, WS and HH gave important input to the manuscript. HH and BH carried out the transmission electron microscopy experiments. All authors read and approved the final manuscript.
